# α-Tocotrienol Protects Neurons by Preventing Tau Hyperphosphorylation via Inhibiting Microtubule Affinity-Regulating Kinase Activation

**DOI:** 10.3390/ijms25158428

**Published:** 2024-08-01

**Authors:** Yuhong Liu, Yunxi Chen, Koji Fukui

**Affiliations:** 1 Molecular Cell Biology Laboratory, Department of Functional Control Systems, Graduate School of Engineering and Science, Shibaura Institute of Technology, Saitama 337-8570, Japan; nb21111@shibaura-it.ac.jp; 2 Molecular Cell Biology Laboratory, Department of Systems Engineering and Science, Graduate School of Engineering and Science, Shibaura Institute of Technology, Saitama 337-8570, Japan; mf22087@shibaura-it.ac.jp

**Keywords:** molecule, Western blotting, cell culture, Alzheimer’s disease, N1E-115, tau hyperphosphorylation, α-tocotrienol, vitamin E, microtubule affinity-regulating kinase

## Abstract

In the pathological process of Alzheimer’s disease, neuronal cell death is closely related to the accumulation of reactive oxygen species. Our previous studies have found that oxidative stress can activate microtubule affinity-regulating kinases, resulting in elevated phosphorylation levels of tau protein specifically at the Ser262 residue in N1E-115 cells that have been subjected to exposure to hydrogen peroxide. This process may be one of the pathogenic mechanisms of Alzheimer’s disease. Vitamin E is a fat-soluble, naturally occurring antioxidant that plays a crucial role in biological systems. This study aimed to examine the probable processes that contribute to the inhibiting effect on the abnormal phosphorylation of tau protein and the neuroprotective activity of a particular type of vitamin E, α-tocotrienol. The experimental analysis revealed that α-tocotrienol showed significant neuroprotective effects in the N1E-115 cell line. Our data further suggest that one of the mechanisms underlying the neuroprotective effects of α-tocotrienol may be through the inhibition of microtubule affinity-regulated kinase activation, which significantly reduces the oxidative stress-induced aberrant elevation of p-Tau (Ser262) levels. These results indicate that α-tocotrienol may represent an intriguing strategy for treating or preventing Alzheimer’s disease.

## 1. Introduction

It is widely recognized that aging plays a significant role in the progression of Alzheimer’s disease (AD) and Parkinson’s disease, which are among the most prevalent neurodegenerative conditions [1,2,3,4]. AD is characterized by decreased learning ability, memory function, and behavioral changes [5]. However, our understanding of the specific mechanisms underlying AD-related cognitive decline remains limited. Based on the studies now available, researchers have observed that autopsies conducted on individuals with AD have shown a notably higher accumulation of neurofibrillary tangles and amyloid plaques compared to the elderly without the disease. In addition, hyperphosphorylated tau protein that forms neurofibrillary tangles and the beta-amyloid (Aβ) that forms amyloid plaques are the main pathological characteristics of AD [6,7]. Recent research has disclosed the presence of a modest level of hyperphosphorylated tau within the cerebral tissue of healthy elderly individuals. However, based on the well-established Braak staging system, this phenomenon rarely progresses beyond Braak stage IV in the context of physiological aging [8]. Thus, the level of tau hyperphosphorylation serves as a key indicator for assessing the progression of AD and its pathological implications.

In Dr. Harman’s free radical theory, reactive oxygen species (ROS), particularly superoxide and hydroxyl radicals, are widely recognized as highly reactive molecules that, when accumulated excessively within organisms, can induce widespread oxidative damage [9]. The human brain utilizes 20% of the body’s oxygen supply, and this significant energy consumption renders neurons, the fundamental functional components of the brain, susceptible to damage from oxidative stress [10]. According to existing research findings, blood indicators of oxidative stress, including 8-hydroxydeoxyguanosine (8-OHdG), 8-hydroxyguanosine (8-OHG), malondialdehyde (MDA), protein carbonyls, and 3-nitrotyrosine, have been demonstrated in people with AD and AD animal models [11,12,13]. This evidence indicates that oxidative stress is a notable pathogenic feature of AD. Furthermore, oxidative stress results in the accumulation of Aβ and hyperphosphorylation of tau, suggesting that oxidative stress plays a significant contributing role in the development of AD [14,15]. Reactive oxygen species (ROS) and free radicals cause the oxidation of vital components in living organisms, including deoxyribonucleic acid (DNA), lipids, and proteins. When DNA undergoes oxidation, the nucleotide 2’-deoxyguanosine, which is a constituent of DNA, forms into 8-hydroxy-2’-deoxyguanosine (8-OHdG) [16]. Mecocci, P et al. observed that there are notably elevated levels of 8-OHdG in mitochondrial DNA within the brains of individuals with AD compared to those without AD [17]. Furthermore, oxidation leads to the occurrence of DNA strand breakage, and a notable increase in the levels of DNA strand breakage was observed in the brains of individuals with AD compared to the control group [18]. Lipids undergo oxidation and involve a series of reactions to form lipid peroxide (LOOH). Once a lipid is oxidized, the oxidation reaction continues in a chain. Furthermore, the oxidation of some unsaturated fatty acids results in the formation of an aldehyde known as 4-hydroxy-2-nonenal (4-HNE). Multiple investigations have shown markedly increased levels of 4-HNE in the brains of patients with AD [19,20]. Many investigations have demonstrated the presence of protein nitration in patients with AD [21]. The nitration of proteins occurs as a result of the reaction between superoxide (O_2_^−^) and nitric oxide (NO), leading to the formation of peroxynitrite (ONOO^−^), and peroxynitrite has more capacity for oxidation [22]. ONOO^−^ reacts with protein tyrosine residues to generate 3-nitrotyrosine (3-NT) [23]. Neurons, integral to the central nervous system, face substantial harm or death when subjected to assault by ROS, posing challenges for brain function recovery. Identifying early signals of neuronal alterations before the advent of cell death is imperative to avert ROS-induced damage to neurons [24].

The aggregation and hyperphosphorylation of tau protein result from the interplay of numerous factors, among which oxidative stress emerges as a significant contributor. Furthermore, the aggregation of Aβ is also believed to be triggered by oxidative stress [13,14]. Aβ peptides are generated by the process of hydrolysis of amyloid precursor protein (APP), which is a transmembrane protein with a single channel. This hydrolysis is performed by β-secretase (BACE1) and γ-secretase, resulting in the production of the Aβ1-40/42 polypeptide. These polypeptides form extracellular Aβ oligomers that are highly cytotoxic [25]. Oxidative stress induces an increase in the expression of APP. Additionally, the activity of BACE1 can be upregulated by oxidative stress, which can further enhance the level of Aβ as an APP product [26]. Multiple lines of evidence indicate that the Aβ peptide itself has a role in causing an increase in ROS and subsequently triggers oxidative stress [27,28,29]. For instance, Aβ oligomers have the ability to directly enhance the formation of ROS by activating NADPH oxidase [30]. Furthermore, the interaction of Aβ with metal ions, such as copper (Cu(I/II))-Aβ and iron (Fe(II/III))-Aβ complexes, contributes to ROS generation through the Fenton reaction, a well-established process that catalyzes the conversion of hydrogen peroxide into hydroxyl radicals, further amplifying oxidative stress within the cellular milieu [31]. This process creates a self-perpetuating cycle between oxidative stress and the aggregation of Aβ. Nevertheless, the clinical outcomes of the treatment strategies targeting Aβ thus far have been unsatisfactory in terms of decreasing Aβ generation, inhibiting Aβ aggregation, and enhancing Aβ clearance. For instance, BACE1 inhibitors have been found to effectively decrease Aβ levels in cerebrospinal fluid. However, they do not have a significant impact on lowering cognitive or functional decline. In fact, certain experimental results even indicate that they may worsen cognitive degradation [32,33]. Several Aβ aggregation inhibitors that have garnered significant interest have been shown to lack clinical effectiveness in clinical trials, despite promising outcomes in preclinical investigations [34,35]. As a therapeutic intervention to promote Aβ clearance, while experimental evidence shows that current mainstream Aβ antibodies can lower cerebrospinal fluid biomarker levels and Aβ plaques, they have not demonstrated clinical effectiveness [36,37,38]. It is precisely because the clinical studies’ lack of shown effectiveness has led academics to be dubious of the technique, which has subsequently increased the focus on the pathology of tau proteins. Tau protein is an unfolded and highly soluble protein that under certain conditions can transition from its monomeric form to oligomers, ultimately leading to the formation of paired helical filaments (PHFs). These PHFs gradually accumulate within nerve cells, facilitating the emergence and progression of neurofibrillary tangles (NFTs). The aggregation of NFTs further exacerbates neuronal damage, contributing significantly to the pathology of neurodegenerative diseases [39,40]. Tau proteins contain multiple phosphorylation sites, current evidence suggests that phosphorylation at Ser262 and Ser356 sites interferes with the regular interaction between tau and microtubules, resulting in the disruption of microtubule assembly and axonal transport mechanisms [41,42,43].

Members of the Ser/Thr protein kinase family, such as microtubule affinity-regulating kinases (MARKs), play crucial roles in modulating the stability of microtubule-associated proteins (MAPs), intracellular signaling mechanisms, cell division processes, and the pathogenesis of AD [44]. The MARK family comprises four proteins: MARK1, MARK2 (also known as EMK1), MARK3 (alternately referred to as C-TAK1), and MARK4 (or MARKL-1) [45]. The phosphorylation of Ser262 in tau results in the impairment of microtubule reassembly, and Ser262 stands out as a key site specifically targeted for phosphorylation by MARKs [46]. In our prior investigation, we provided evidence that oxidative stress had a substantial impact on the hyperphosphorylation of tau at Ser262. Additionally, we observed that oxidative stress concurrently facilitated a notable increase in MARK activation [47].

Vitamin E is a widely occurring fat-soluble vitamin in nature, renowned for its exceptional antioxidant properties. Furthermore, vitamin E also plays a role in anti-aging processes [48]. Vitamin E mainly comprises two subtypes: tocopherols (Tocs) and tocotrienols (T3s). The distinction between these two subtypes lies in the presence or absence of a double bond in the side chain connecting to the chromophore. Further subdivision reveals that each subtype encompasses four isoforms: α, β, γ, and δ. The differences among these isoforms are determined by the number and position of methyl groups attached to the chroman ring [49]. A number of studies have shown that vitamin E exhibits additional benefits such as anti-inflammatory [26], anti-cancer [27], and neuroprotective properties [50], among others. Among the different isoforms of vitamin E, T3s have garnered significant interest owing to their putative neuroprotective properties [51,52,53,54,55]. Several pieces of evidence have shown that α-tocotrienol (α-T3) has the most potent neuroprotective capacity within the vitamin E family [24]. Therefore, we suggest that exploring the underlying mechanism of the neuroprotective effect of α-T3 holds considerable academic importance.

The objective of this research is to assess the potential neuroprotective effects of α-T3 in inhibiting the hyperphosphorylation of tau protein at Ser262 site, and to evaluate whether this is linked to the activation of MARKs. Through this investigation, we aim to gain a deeper insight into how α-T3 might exert its neuroprotective effects and contribute to the development of more effective therapeutic strategies for neurodegenerative diseases.

## 2. Results

### 2.1. Optimization of α-Tocotrienol Concentration

**Figure 1:**
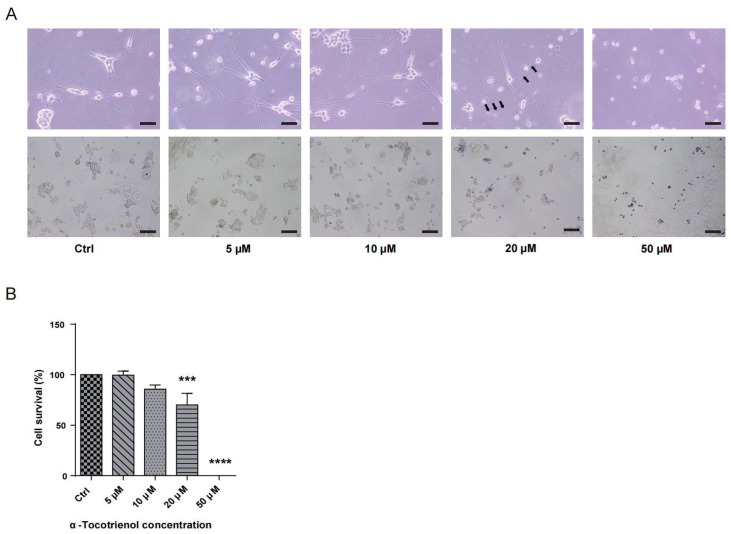
Cytotoxic effects of α-T3 on N1E-115 cells. (**A**) N1E-115 cell morphology was examined following exposure to different doses of α-T3. Control (Ctrl), without α-T3 exposure. Scale bar: 200 μm (**above**). Black arrows indicate neurite deformation. N1E-115 cell morphology following exposure to α-T3. Cells were stained with trypan blue. Scale bar: 50 μm (**below**). (**B**) Cell survival following exposure to different doses of α-tocotrienol was evaluated using trypan blue staining. α-T3 showed a concentration-dependent effect on N1E-115 cell death. The survival rate in the control group, without α-T3, was defined as 100%. The data are shown graphically as the mean value ± SD of three independent experiments (*n* = 3). The statistical significance levels are denoted as follows: *** *p* < 0.001, **** *p* < 0.0001 (two-tailed one-way ANOVA with Tukey’s multiple comparisons test).

To determine the neuroprotective potential of α-T3, it was essential to identify its optimal concentration. N1E-115 cells were subjected to varying concentrations of α-T3 (0, 5, 10, 20, and 50 μM) and cell viability was assessed. Figure 1A illustrates the deformation, attributed to α-T3 cytotoxicity, and depicts the morphology of deceased cells following trypan blue staining. Notably, treatment with >10 μM α-T3 resulted in significant cell death. At concentrations >10 μM, a significant amount of neurite deformation became apparent, as shown by black arrows (Figure 1A, above). The number of live cells (unstained) exhibited a marked decrease at concentrations greater than 10 μM α-T3 (Figure 1A, below). Compared to the control, cell survival experienced a significant reduction at α-T3 concentrations >10 μM (*** *p* < 0.001, **** *p* < 0.0001; Figure 1B). With increasing concentrations, both cell deformation and the proportion of cell death increased following exposure to elevated α-T3 concentrations. Elevated concentrations of α-T3 might induce cytotoxic effects, leading to neuronal degeneration and cell death. Consequently, antioxidant and non-cytotoxic effects may only be achievable at concentrations that do not exceed 10 μM.

### 2.2. Neurite Degeneration and the Neuroprotective Effect of α-Tocotrienol

**Figure 2:**
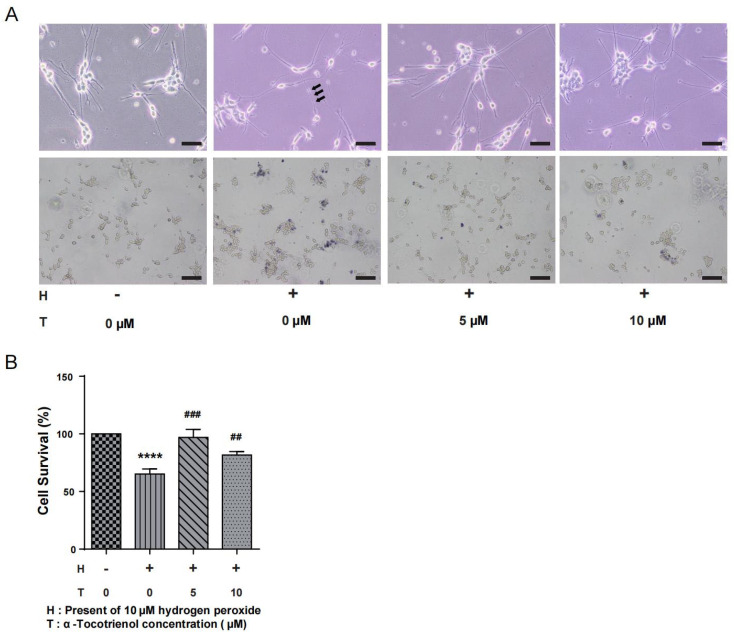
Neuroprotective effects of α-T3 in N1E-115 cells. (**A**) Neurite degeneration was induced in N1E-115 cells by exposure to 10 μM hydrogen peroxide. The neuroprotective effect of different concentrations of α-T3 was observed. The group absent of both hydrogen peroxide and T3 serves as the negative control. Scale bar: 200 μm (**above**). Neuronal deformation is shown by black arrows. Cell morphology of N1E-115 cells was examined using trypan blue staining following co-treatment with different doses of α-T3 and 10 μM hydrogen peroxide. Scale bar: 50 μm (**below**). (**B**) Cell survival was evaluated by trypan blue staining after exposure to different doses of α-T3 with/without 10 μM hydrogen peroxide. The survival rate in the control group, without hydrogen peroxide and α-T3, was set at 100%. The data are represented as mean ± SD of three independent experiments (*n* = 3). The statistical significance levels are denoted as follows: **** (*p* < 0.0001) compared with the control group, ## (*p* < 0.01) and ### (*p* < 0.001) compared with the hydrogen peroxide exposure group (two-tailed one-way ANOVA with Tukey’s multiple comparisons test).

N1E-115 cells were subjected to different doses of α-T3 (0, 5, and 10 µM) to investigate its potential neuroprotective effects in the presence of oxidative stress. Simultaneously, exposure of the cells to 10 µM hydrogen peroxide led to the degeneration of neurites, a consequence of the induced oxidative stress. The group exposed to hydrogen peroxide, as seen in Figure 2A (above), had distinct neuronal beading, which is a reliable indicator of neuronal damage. α-T3 at concentrations of 5 and 10 µM resulted in significant prevention of hydrogen peroxide-induced neurite degeneration (Figure 2A, above). Trypan blue staining, as seen in Figure 2A (below), revealed a significant decrease in the number of viable cells (unstained) following exposure to hydrogen peroxide. The only group that showed a significant decrease in cell survival rate compared to the control group was the one treatment with 10 µM hydrogen peroxide (**** *p* < 0.0001, Figure 2B). In addition, the group exposed to 5 µM α-T3 exhibited a significant increase in cell survival as compared to the group only exposed to hydrogen peroxide (## *p* < 0.01, ### *p* < 0.001, Figure 2B).

### 2.3. α-Tocotrienol Reduces Phosphorylation of Tau at Ser262 under Oxidative Stress

**Figure 3:**
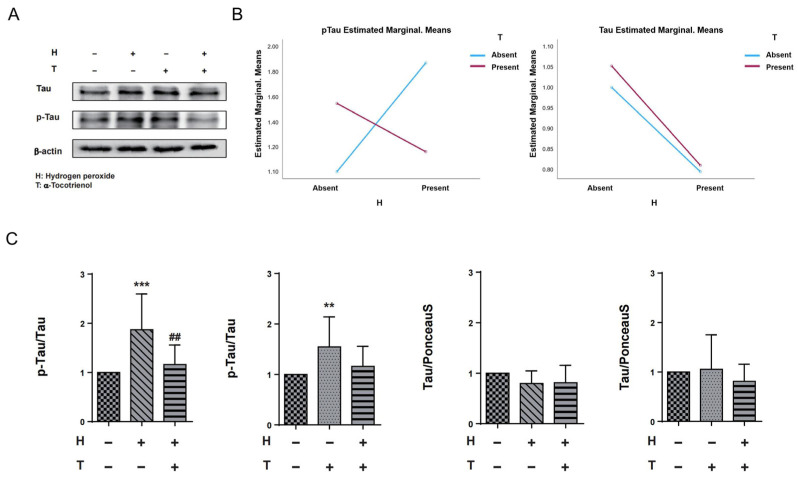
Expression of tau and p-Tau in N1E-115 cells following α-T3 and hydrogen peroxide treatment. (**A**) Western blot analyses were performed to examine the levels of tau and p-Tau in N1E-115 cells with and without hydrogen peroxide and/or α-T3. Representative gels are shown. β-actin was used to visually confirm that similar amounts of protein were loaded. (**B**) The results of two-way ANOVA are shown to illustrate the differences in changes in p-Tau and tau expression induced by hydrogen peroxide and α-T3. The *Y*-axis represents the estimated marginal means effect of the reagents on p-Tau and tau. Data were analyzed using two-way ANOVA with hydrogen peroxide (with and without) and α-T3 (with and without) as factors (*p* < 0.001). (**C**) Changes in tau and p-Tau (Ser262) levels in N1E-115 cells following single or combination exposure to hydrogen peroxide and α-T3. Each column indicates the average protein levels relative to the control. The ratios of tau protein band intensity to Ponceau S staining intensity are shown, and p-Tau band intensity to tau intensity; the ratios of the control samples are defined as 1. The level of p-Tau was divided by that of unphosphorylated tau. Data are plotted as the mean ± SD of three independent experiments (*n* = 12). ** (*p* < 0.01) compared with the ctrl group, ## (*p* < 0.01) compared with hydrogen peroxide exposure group, *** (*p* < 0.001) compared with the ctrl group (two-tailed one-way analysis of variance (ANOVA) with Tukey–Kramer’s test).

In order to assess the potential interaction effect between hydrogen peroxide and α-T3, we conducted a two-way ANOVA analysis with hydrogen peroxide and α-T3 as fixed variables, both with and without their respective additions. Results of the two-way ANOVA indicated that there was no interaction effect between hydrogen peroxide and α-T3 for tau measurements. However, a significant interaction effect was observed between hydrogen peroxide and α-T3 for p-Tau measurements (*p* < 0.001; Figure 3B, left panel). To ascertain whether the neuroprotective effect of α-T3 is mediated through the disruption of hyperphosphorylation of tau proteins at Ser262 in response to oxidative stress, we confirmed the effects of α-T3 on tau and p-Tau, respectively, by a one-way ANOVA based on fixed factor α-T3 and fixed factor hydrogen peroxide, respectively. Compared to the control group, cells exposed to hydrogen peroxide exhibited a significant increase in phosphorylated tau protein (Ser262) levels (*** *p* < 0.001; Figure 3C, first from left). However, in cells treated with both hydrogen peroxide and α-T3, there was a notable decrease in phosphorylated tau (Ser262) levels compared to cells exposed to hydrogen peroxide alone in the absence of α-T3 (## *p* < 0.01; Figure 3C, first from left). Interestingly, cells exposed to α-T3 alone also demonstrated a significant elevation in phosphorylated tau protein (Ser262) levels compared to the control group (** *p* < 0.01, Figure 3C, second from left). On the contrary, irrespective of the fixed factor being hydrogen peroxide or α-T3, no notable difference in tau protein expression was observed between the experimental and control groups (ns; Figure 3C, third and fourth from left).

### 2.4. α-Tocotrienol Inhibits Tau Phosphorylation at Ser262 via MARK Pathway Activation

**Figure 4:**
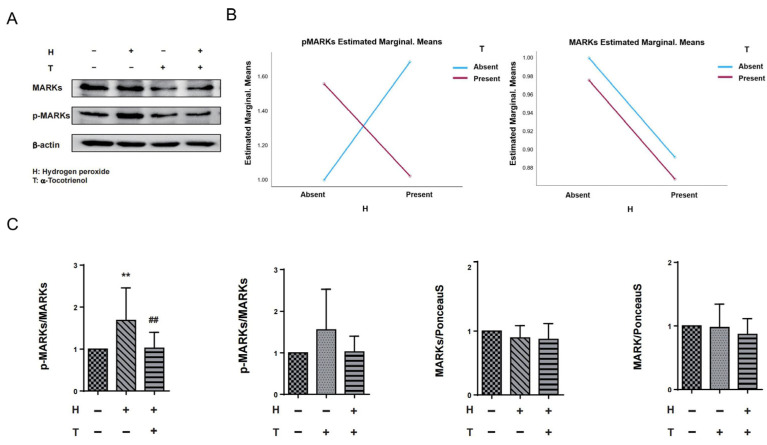
Expression levels of MARKs and p-MARKs in N1E-115 cells following α-T3 and hydrogen peroxide exposure. (**A**) Western blot analysis was performed to examine the levels of MARKs and p-MARKs in N1E-115 cells with and without hydrogen peroxide and/or α-T3. Representative gels are shown. β-actin was used to visually confirm that similar amounts of protein were loaded. (**B**) The results of two-way ANOVA are shown to illustrate the differences in changes in MARK and p-MARK expression induced by hydrogen peroxide and α-T3. The *Y*-axis represents the estimated marginal means effect of the reagents on p-Tau and tau. Data were analyzed using two-way ANOVA with hydrogen peroxide (with and without) and α-T3 (with and without) as factors (*p* = 0.002). (**C**) Changes in MARK and p-MARK levels in N1E-115 cells following single or combination exposure to hydrogen peroxide and α-T3. Each column indicates the average protein levels relative to the control. The ratios of each protein band intensity to Ponceau S staining intensity are shown; the ratios of the control samples are defined as 1. The level of p-MARKs was divided by that of the unphosphorylated MARKS. Data are plotted as mean ± SD of three independent experiments (*n* = 12). ** (*p* < 0.01) compared with the ctrl group, ## (*p* < 0.01) compared with the hydrogen peroxide exposure group (two-tailed one-way analysis of variance (ANOVA) with Tukey–Kramer’s test).

The results of two-way ANOVA indicated that there was no interaction between hydrogen peroxide and α-T3 on MARKs. However, a significant interaction effect was observed between hydrogen peroxide and α-T3 for p-MARKs (*p* = 0.002; Figure 4B, left panel). In order to investigate the potential neuroprotective effects of α-T3, its implication on tau hyperphosphorylation at Ser262 via MARK activation was examined by evaluating the levels of MARKs and p-MARKs by Western blotting. We confirmed the effects of α-T3 on MARKs and p-MARKs, respectively, by a one-way ANOVA based on α-T3 and hydrogen peroxide as fixed factors, respectively. With hydrogen peroxide as the fixed factor, cells treated with hydrogen peroxide exhibited levels of p-MARKs that were significantly increased compared to the control group (** *p* < 0.01; Figure 4C, first from left). In comparison, the cells that were simultaneously exposed to hydrogen peroxide and α-T3 demonstrated a notable decrease in p-MARK levels when compared to the cells that were not treated with α-T3 (## *p* < 0.01; refer to Figure 4C, first from left). With α-T3 as a fixed factor, there were no significant differences in the expression of p-MARKs between the experimental and control groups (ns; Figure 4C, second from left). Regardless of whether hydrogen peroxide or α-T3 was used as the constant factor, our analyses revealed no substantial variations in MARK expression between the experimental and control groups (ns; Figure 4C, third and fourth from left).

## 3. Discussion

### 3.1. High Concentrations of α-Tocotrienol Exhibit Cytotoxic Effects on N1E-115 Cells

Our previous investigation showed that exposure to 10 μM hydrogen peroxide leads to the degeneration of neurites [47]. This evidence supports a correlation between oxidative stress and neurological impairment in N1E-115 cells. Hence, we assumed that it may be achievable to reduce the impact of oxidative stress on nerve cells by using the neuroprotective features of a particular compound. We performed an experiment using α-T3, a compound known for its outstanding antioxidant properties and neuroprotective effects in different cell lines [24,52,53]. We first evaluated the toxicity of α-T3 in the N1E-115 cell line. The results indicate that at an α-T3 concentration >10 μM, neurite degeneration becomes apparent (Figure 1A, indicated by black arrows), and cell death is observed (Figure 1B). Statistical analysis of the results revealed a significant difference in cell viability at concentrations >10 μM. At concentrations of 50 μM, cell survival decreases further, approaching zero. The results presented here indicate that N1E-115 cells exposed to the negative impacts of α-T3 show cytotoxicity at concentrations above 10 μM. Currently, there are no studies available that explain the specific mechanism by which α-T3 causes cytotoxicity in N1E-115 cells. However, several investigations have presented us with potential reasons. Some evidence suggests that γ-T3 caused apoptosis via regulating mitochondrial activity [52]. δ-T3 inhibits the proliferation of human pancreatic cancer cells and induces apoptosis through the activation of caspase-8 and caspase-3, both in vitro and in vivo [56]. Research in ovarian cancer cells showed that treatment with δ-T3 resulted in G1 cell cycle arrest and indicated that δ-T3 induced apoptosis by producing the release of cytochrome c from mitochondria [57]. All of these data indicate a positive relationship between various types of vitamin E and apoptosis. Poly (ADP-ribose) polymerase (PARP) is an enzyme that aids in repairing DNA damage by forming poly (ADP-ribose) chains to mend DNA and maintain its integrity when DNA is damaged [58]. When irreversible damage occurs to DNA, PARP regulates apoptosis through specific cleavage [59]. According to Loganathan et al., α-, δ-, and γ-T3 have the ability to induce apoptosis in cancer cells by blocking or inhibiting PARP [59]. In addition, α-, δ-, and γ-T3 exert inhibitory effects on cancer cells by inhibiting the activity of nuclear factor kappa-B (NF-κB), which facilitates the proliferation of cancer cells [60]. Furthermore, Li et al. revealed that the cleavage of PARP was among the mechanisms responsible for inducing apoptosis in N1E-115 cells [60]. This evidence provides a possible explanation for our current research findings. We hypothesize that α-T3 may exert cytotoxic effects on N1E-115 cells by cleaving or inhibiting PARP, ultimately triggering apoptosis. However, due to the lack of apoptosis detection in this study, further research is needed to elucidate the underlying mechanism.

### 3.2. Neuroprotective Effects of α-Tocotrienol

Following experimental confirmation, we determined that α-T3 exposure at concentrations below 10 µM did not exhibit overt cytotoxicity in N1E-115 cells (as shown by cell survival rate). Next, we examined the neuroprotective properties of α-T3 at low doses. The results indicate that 10 µM hydrogen peroxide produces neurodegeneration in the N1E-115 cells, as shown by the black arrow in Figure 2A. The neurodegeneration was greatly ameliorated by the presence of 5 and 10 µM α-T3. Our trypan blue staining results revealed a significant decrease in the survival rate after hydrogen peroxide treatment. In the group exposed to both hydrogen peroxide and α-T3, there was a significant increase in the survival rate for both 5 and 10 µM α-T3, compared to the group with hydrogen peroxide but without α-T3 (Figure 2A unstained, Figure 2B). These findings confirmed that α-T3 has a neuroprotective effect on N1E-115 cells by preventing the degeneration of neuronal protrusions following hydrogen peroxide exposure and reducing cell death. This suggests that α-T3 is beneficial for protecting neuron-like N1E-115 cells. Numerous studies have investigated the neuroprotective functions of α-T3. α-T3 exhibited the highest level of neuroprotection among the various isoforms of vitamin E in research conducted using rat striatal cultures [61]. However, based on evaluations of protein oxidation and lipid peroxidation, γ-T3 had the highest efficacy, followed closely by α-T3 [62]. The neuroprotective properties of α- and γ-T3 did not exhibit a direct link with their relative antioxidant activity, indicating that the neuroprotective effects of α-T3 may not be exclusively attributed to its free radical-scavenging ability, i.e., antioxidant capacity. A study reported that α-T3 demonstrated antioxidant and neuroprotective properties within the concentration range of 5 to 50 µM [63]. Moreover, exposure to a low concentration range (100–250 nM) did not exhibit antioxidant properties; however, it inhibited the increase in levels of calcium (Ca^2+^) generated by glutamate during the early phases of apoptosis. Thus, the low concentrations of α-T3 may prevent apoptosis by inhibiting the release of Ca^2+^. Other studies have shown similar results, with α-T3 exerting neuroprotective effects through antioxidant properties at high concentrations [64,65]. On the other hand, cPLA2 is an enzyme that induces the release of free fatty acids from phospholipids within cells. This process frees arachidonic acid (AA) from membrane phospholipids, and the presence of free AA induces apoptosis [66]. Furthermore, cPLA2 is triggered by the presence of Ca^2+^. Research has shown that when α-T3 is present at nanomolar concentrations, the alterations in cytosolic phospholipase A2 (cPLA2) caused by glutamate represent a neuroprotective mechanism of α-T3 besides the antioxidant effects [67]. Taken together, α-T3 at high concentrations exerts neuroprotection through antioxidant properties and at low concentrations through mechanisms that are not dependent on antioxidant properties, such as inhibition of cPLA2. However, in the present study, it is unclear whether the concentration of α-T3 used provides neuroprotective effects in N1E-115 cells via antioxidant-dependent, antioxidant-independent, or a combination of these mechanisms. Additional investigation is required to clarify this matter.

### 3.3. α-Tocotrienol May Have a Neuroprotective Effect by Inhibiting the Phosphorylation of Tau at Ser262

The experimental findings showed that α-T3 has neuroprotective effects in N1E-115 cells at concentrations of 5 and 10 µM (Figure 2B), as shown by cell survival rate. The results of Western blot analysis revealed that α-T3 significantly decreased the tau hyperphosphorylation induced by oxidative stress (*p* < 0.01; Figure 3C, first from left). We hypothesize that the observed inhibition of tau hyperphosphorylation indicates a positive relationship with the neuroprotective effect of α-T3. Nevertheless, as previously stated, the neuroprotective mechanisms of α-T3 may be separated into antioxidant and non-antioxidant mechanisms. Figure 3B displays the findings of two-way ANOVA (*p* < 0.001), which revealed an inverse relationship between hydrogen peroxide and α-T3. These findings indicate that the ability of α-T3 to prevent abnormal tau hyperphosphorylation may be attributed to its antioxidant properties. Although there is no direct evidence for a mechanism by which α-T3 reduces the high tau hyperphosphorylation, some research conducted on vitamin E provides possible explanations for our present experiment. Preliminary studies suggest that overexpressing antioxidant enzymes like mitochondrial superoxide dismutase (SOD2) or exposing cells to vitamin E can attenuate tau-induced neuronal death. Conversely, a decrease in SOD2 levels or a reduction in cytoplasmic SOD (SOD1) appears to enhance tau phosphorylation [68,69,70]. This is consistent with our findings that α-T3 may also reduce tau phosphorylation through its antioxidant properties. Furthermore, it was shown that tau-induced neurodegeneration in *Drosophila* is decreased by treatment with vitamin E [71]. Furthermore, several animal models have also demonstrated that vitamin E reduces tau phosphorylation and improves neurodegeneration. Supplementation of tau transgenic mice with α-tocopherol was demonstrated to suppress/delay the progression of tau pathology, improve function, and reduce behavioral symptoms which are defined as dystonic movements of the hind limbs or a combination of hind limbs, forelimbs, and trunk, in which the limbs are retracted towards the body in a way not seen in normal mice. In another in vitro study, α-T3 showed inhibitory effects at a dose of 10 μM on Aβ accumulation, which is a hallmark of AD [72]. However, whether Aβ buildup is a cause or a result of other pathogenic processes in AD remains a subject of debate [73,74]. Interestingly, in addition to the inhibitory effect of α-T3 on the phosphorylation of tau, our experiments also revealed a significant increase in the hyperphosphorylation of tau in the α-T3 group (** *p* < 0.01; Figure 3C, second from left). There is yet limited evidence of the direct effect of α-T3 on the phosphorylation of tau. Nonetheless, there are indications of circumstantial evidence that suggest potential underlying mechanisms at play. α-T3 has been demonstrated to exhibit a pro-apoptotic effect [75]. As the process of apoptosis necessitates the excessive accumulation of Ca2+ within the mitochondria [75,76]. Calcium/Calmodulin-dependent protein kinase II (CaMKII) becomes activated in response to elevated concentrations of Ca2+ [77,78]. Research has indicated that CaMKII is capable of phosphorylating tau at the Ser262 site [79]. However, neither the activation of CaMKII nor alterations in intracellular calcium concentration were evaluated within the scope of our current investigation. By incorporating indirect evidence pertaining to these mechanisms, we assume that the induction of apoptosis by α-T3 results in an increase in Ca2+ levels, hence activating the activation of CaMKII. This offers a convincing explanation for the observation that the α-T3-added group exhibited notably elevated levels of p-Tau expression. Drawing upon the comprehensive discussion of these data, α-T3 can effectively prevent the abnormal phosphorylation of tau, which is compelling evidence for the significant therapeutic and preventive potential of α-T3 for treating AD.

### 3.4. α-Tocotrienol May Play a Role in Inhibiting the Activation of MARKs

The MARK family consists of four proteins. The primary antibody utilized in our study encompassed four isoforms of MARKs as well as four distinct isoforms of p-MARKs phosphorylated at various sites. In our earlier investigation, we observed a significant increase in p-MARK expression levels under conditions of oxidative stress [47]. In our current investigation, we have obtained similar results. The introduction of hydrogen peroxide resulted in a significant increase in p-MARK expression levels (** *p* < 0.01; Figure 4C, first from left). As previously stated, the current research does not provide any data on the specific mechanism by which α-T3 was significantly decreasing tau hyperphosphorylation. In our current research, we noticed a significant decrease in the increase in p-MARKs caused by oxidative stress when α-T3 was applied (## *p* < 0.01; Figure 4C, first from left). This finding provides evidence that α-T3 performs its effect by reducing tau hyperphosphorylation via the MARK–tau signaling pathway. Nevertheless, to date, no research has been documented elucidating the precise mechanism underlying the neuroprotective effects of α-T3 through its interaction with MARKs. Although our current study’s data indeed point to the influence of α-T3 on MARK expression levels, the aforementioned constraints posed by the primary antibodies employed have hindered our ability to discern the specific MARK isoforms that underwent activation or inactivation. Furthermore, we were unable to ascertain which isoform exerted the most profound impact on the phosphorylation of tau protein at the Ser262 site. As we have previously highlighted, tau phosphorylation at the Ser262 site is intricately involved not only in the initiation of early tau lesions but also in the subsequent aggregation, seeding, and progression of AD pathology [80,81]. The mechanisms by which the Ser262 site of tau undergoes phosphorylation are highly deserving of thorough research and extensive discussion. There is some evidence that shows that all MARK isoforms are involved in tau phosphorylation at Ser262, and MARK4 appears to play a more important role in this process than other MARK isoforms [82]. A recent study has revealed intriguing insights that different isoforms of the MARK family do not work the same way for tau; researchers indicate that in a *Drosophila* model, MARK1 and MARK2 increase the phosphorylation of the Ser262 site of tau protein, with MARK4 not only enhancing the phosphorylation at the Ser262 site but also elevating the overall expression levels of tau in vivo [83]. Moreover, in vivo experiment, MARK4 exhibits the capacity to engage in interactions with other crucial tau kinases, including cyclin-dependent kinase 5 (Cdk5), for example, which could potentially modulate the extent or intensity of Ser262 phosphorylation, thereby contributing to an even more intricate regulatory network surrounding tau phosphorylation [84]. In addition, several studies have indicated that MARK4 is crucial to the pathophysiology of several diseases, including AD [85,86]. While the intricate mechanisms underpinning the specific isoforms are not delved into within the scope of our current study, we posit that MARK4 harbors a more elaborate and nuanced mechanism for the phosphorylation of Ser262, hinting at a complexity yet to be fully unraveled.

On the other hand, we have yet to conduct further investigations of the interplay between oxidative stress and α-T3 on each MARK isoform. Investigations have indicated that MARK4 overexpression aggravated oxidative stress by increasing ROS activity and reducing the activities of catalase (CAT), glutathione peroxidase (GPx), SOD, and reduced and oxidized glutathione (GSH/GSSG) [87,88]. In addition, researchers also confirmed that although MARK4 dramatically reduced mitochondrial oxidative respiration and increased oxidative stress, treatment with vitamin E reduced this impact [89]. Moreover, the present study has not ventured into exploring the mechanism of α-T3’s action on MARKs at this juncture. Since MARKs play an influential role in cancer as well, there may also be an effect by other signaling pathways of α-T3 on MARK action due to the anti-cancer mechanism of α-T3. There are pertinent findings that demonstrate the significant impact of oxidative stress on cancer. ROS have been reported to alter the expression of the apoptosis-critical gene p53, which is a suppressor gene [90]. On the other hand, the expression of MARK2, which is one isoform of the MARK family, has been reported to be negatively correlated with p53 [91]. p53 is a protein that can be activated by a variety of stressors, including DNA damage, oncogene activation, and hypoxia, and activation of p53 inhibits the proliferation of injured cells via apoptosis [92]. Some researchers have proposed that the anti-cancer effect of α-T3 was achieved by inducing apoptosis [60].

Taken together, it is also particularly valuable to investigate the effect of oxidative stress and α-T3 on the different MARK family isoforms. The levels of MARK4 protein were not evaluated in this study, and therefore, additional research is required to address this gap. In conclusion, determining the precise impact of α-T3 on each MARK isoform merits attention. The findings of this study have provided us with numerous ideas for further research directions. For instance, we can delve into the expression changes of various isoforms of MARKs upon the addition of α-T3, or investigate whether α-T3-induced apoptosis is also associated with different isoforms of MARKs.

## 4. Materials and Method

### 4.1. Cell Culture

The cell culture method remained consistent with the previously stated procedure [47]. In summary, the N1E-115 cell line was initially incubated in Dulbecco’s minimal medium for 24 h, and subsequently shifted to a serum-free conditioned medium enriched with 1% dimethylsulfoxide (DMSO) to stimulate neurite outgrowth. Cells exhibiting neurite elongation were then further incubated for 48 h to prepare them for subsequent experimental procedures. Post-incubation, cells were seeded in 35 mm dishes at a density of 4 × 10^4^ cells/mL for the staining experiment and in 60 mm dishes at a density of 70 × 10^4^ cells/mL for protein extraction. The conditioned media utilized in this research were collected from N1E-115 cells cultured for 48 h until 80–90% confluency was reached. Notably, fetal bovine serum (FBS) was intentionally excluded during the cultivation process, and 1% dimethyl sulfoxide (DMSO) was added to the medium. This uniquely prepared medium was shown to efficiently promote neurite elongation.

### 4.2. Optimization of α-Tocotrienol Concentration

To determine the optimal concentration of α-T3 for our experiments, cell viability was assessed using the trypan blue staining exclusion method. After neurite elongation, cells were treated with different doses of α-T3, ranging from 0 to 50 µM, for a duration of 24 h. Following treatment, each dish was stained with trypan blue solution (final concentration 0.4%) and incubated at 37 °C in a CO_2_ incubator for 20 min. Subsequently, cells were washed with phosphate-buffered saline (PBS) to remove excess dye. For analysis, images of cells treated with different α-T3 concentrations were captured utilizing an Olympus IX81 phase-contrast microscope, which was complemented by an Olympus DP71 digital camera, both sourced from Olympus Corp. in Tokyo, Japan. Live and total cell counts were determined by randomly selecting photomicrographs. The percentage of dead cells was then calculated relative to the total cell number. Each concentration of α-T3 was tested on at least three plates, and triplicate experiments were performed to ensure reproducibility.

### 4.3. Neurite Degeneration

The morphological changes associated with neurite degeneration, including bead formation, were evaluated in our study [93]. For the purpose of determining the morphological changes indicative of neuronal degeneration, simulated oxidative stress was produced by utilizing hydrogen peroxide (10 µM). The protective impact of α-T3 on cells was observed through the co-treatment of various concentrations of α-T3. Cells were fixed using a 4% paraformaldehyde solution (Merck Millipore, Darmstadt, Germany) diluted in PBS at 4 °C for 15 min. Image processing was conducted using an Olympus IX81 phase-contrast microscope coupled with a DP71 digital camera, and images were transferred to a computer for analysis. Each experimental group comprised a minimum of three dishes, and cells were randomly selected for observation within each group. This experimental procedure was repeated three times to ensure the reliability of the findings.

### 4.4. Western Blotting

N1E-115 cells were isolated in RIPA buffer (#182-02451, FUJIFILM Wako Pure Chemical Corp., Osaka, Japan) after being collected from 60 mm cell culture dishes. N1E-115 cell lysate samples were collected and centrifuged, and the protein content of the resulting supernatant was determined using a Bradford assay employing the Bio-Rad Protein Assay Dye Reagent Concentrate (#5000006; Bio-Rad Laboratories, Inc., Hercules, CA, USA) according to the manufacturer’s protocol. Electrophoresis was performed using the same experimental methods as previously described [47]. After electrophoresis, the cell membrane was blocked with a 2% skim milk solution at RT for 1 h, followed by incubation with the primary antibody obtained from Abcam, Cambridge, UK, at 4 °C overnight. We used secondary antibodies for the primary antibodies’ originating species, which were rabbit and mouse (detailed information on primary antibodies and secondary antibodies can be found in the Supplementary Materials, Table S1). Following this, chemiluminescent signals were utilized using experimental procedures in our laboratory [47]. Relative ratios were determined by comparing the intensity of Ponceau S staining in the corresponding lane with the signal intensity of the respective experimental protein. These comparisons were streamlined through the utilization of Image Quant TL 8.1 software, sourced from GE Healthcare Life Sciences in Tokyo, Japan. To ensure reproducibility and reliability, each study encompassed a minimum of three independent experimental runs.

### 4.5. Statistical Analysis

The data were graphically represented as the mean ± SD, derived from a minimum of three independent experiments. Two-way analysis of variance (ANOVA) was applied to explore interactive effects between different drugs. Estimated marginal means were calculated to illustrate predicted means across various factor-level combinations in a two-factor interaction plot using IBM SPSS Statistics Client 29.0 (International business Machines Co., Armonk, NY, USA). Additionally, two-way ANOVA was conducted to evaluate interaction effects between factors and their influence on relative protein expression ratios across different conditions. To compare across three or more groups, a two-tailed one-way ANOVA was applied, subsequently followed by a Tukey–Kramer post hoc analysis using JMP pro.16 software (SAS Institute Japan Co., Ltd., Tokyo, Japan). *p*-values < 0.05 indicated a significant difference. Data were visualized using Prism version 6.02 (GraphPad Software, San Diego, CA, USA).

## 5. Conclusions

Treatment with α-T3 reduced the oxidative stress-induced increases in phospho-tau at Ser262. Furthermore, α-T3 exhibited a neuroprotective effect at low concentrations during oxidative stress (i.e., hydrogen peroxide exposure). This might indicate that the neuroprotective effect of α-T3 may act by preventing tau hyperphosphorylation at Ser262.

The addition of α-T3 significantly reduced the activation of MARKs, suggesting that α-T3’s inhibition of MARK activation may be one of the mechanisms by which it suppresses the hyperphosphorylation of tau proteins. These results indicate that α-T3 could potentially serve as a tau protein phosphorylation inhibitor and may have therapeutic implications as an adjuvant treatment for AD. Furthermore, the study of α-T3 holds significant research value in AD and other medical conditions.

## Data Availability

The datasets used and/or analyzed during the current study are available from the corresponding author upon reasonable request.

## Supplementary Materials

The following supporting information can be downloaded at https://www.mdpi.com/article/10.3390/ijms25158428/s1.
