# Supplementary material for: α-Tocotrienol Protects Neurons by Preventing Tau Hyperphosphorylation via Inhibiting Microtubule Affinity-Regulating Kinase Activation
Source: Int J Mol Sci. 2024 Aug 1;25(15):8428. doi: 10.3390/ijms25158428 (PMC11313320; doi:10.3390/ijms25158428)
Supplement: Supplementary file 1 [file ijms-25-08428-s001.zip › ijms-3089634-supplementary.pdf]

Supplementary information

Antibody details:
Priamry antibodies:
Mouse monoclonal anti-TAU-5 antibody, #ab80579;
Rabbit polyclonal anti-Tau (phospho S262) antibody, #ab131354;
Rabbit polyclonal anti-MARK 1+2+3+4 antibody, #ab74131;
Rabbit monoclonal anti-MARK4 (phospho T214) + MARK2 (phospho T208) + MARK3 (phospho T234) + MARK1 antibody (EPR5463), #ab126731;
Mouse monoclonal anti-β-actin antibody, #ab8226;
Priamry antibodies from Abcam, Cambridge, UK

Secondary antibodies:
Horseradish peroxidase(HRP)-conjugated anti-rabbit IgG antibody,#W4011;
Horseradish peroxidase(HRP)-conjugated anti-mouse IgG antibody,#W4021;
Secondary antibodies from Promega Corp., Madison, WI, USA.

Table S1. Concentration and reaction time of antibodies.

	Antibody	Concentration	Host	Reaction time
Primary antibodies	Anti-tau [5]	1/2000	mouse	Over night
	Anti-pho-tau [S262]	1/2000	rabbit	
	Anti-β-actin	1/4000	mouse	
	Anti-MARKs	1/1000	rabbit	
	Anti-pho-MARKs	1/2000	rabbit	
Secondary antibodies	Anti-Mouse IgG HRP	1/4000	mouse	1h
	Anti-Rabbit IgG HRP	1/4000	rabbit	
